# A Single Crossing-Over Event in Voltage-Sensitive Na^+^ Channel Genes May Cause Critical Failure of Dengue Mosquito Control by Insecticides

**DOI:** 10.1371/journal.pntd.0003085

**Published:** 2014-08-28

**Authors:** Koichi Hirata, Osamu Komagata, Kentaro Itokawa, Atsushi Yamamoto, Takashi Tomita, Shinji Kasai

**Affiliations:** 1 Department of Environmental and Toxicology, Odawara Research Center, Nippon-soda Co., Ltd., Odawara, Kanagawa, Japan; 2 Department of Medical Entomology, National Institute of Infectious Diseases, Shinjuku-ku, Tokyo, Japan; 3 Department of Research and Develop Management, Nippon-soda Co., Ltd., Chiyoda-ku, Tokyo, Japan; Johns Hopkins Bloomberg School of Public Health, United States of America

## Abstract

The voltage-sensitive sodium (Na^+^) channel (Vssc) is the target site of pyrethroid insecticides. Pest insects develop resistance to this class of insecticide by acquisition of one or multiple amino acid substitution(s) in this channel. In Southeast Asia, two major Vssc types confer pyrethroid resistance in the dengue mosquito vector *Aedes aegypti*, namely, S989P+V1016G and F1534C. We expressed several types of Vssc in *Xenopus* oocytes and examined the effect of amino acid substitutions in Vssc on pyrethroid susceptibilities. S989P+V1016G and F1534C haplotypes reduced the channel sensitivity to permethrin by 100- and 25-fold, respectively, while S989P+V1016G+F1534C triple mutations reduced the channel sensitivity to permethrin by 1100-fold. S989P+V1016G and F1534C haplotypes reduced the channel sensitivity to deltamethrin by 10- and 1-fold (no reduction), respectively, but S989P+V1016G+F1534C triple mutations reduced the channel sensitivity to deltamethrin by 90-fold. These results imply that pyrethroid insecticides are highly likely to lose their effectiveness against *A. aegypti* if such a *Vssc* haplotype emerges as the result of a single crossing-over event; thus, this may cause failure to control this key mosquito vector. Here, we strongly emphasize the importance of monitoring the occurrence of triple mutations in *Vssc* in the field population of *A. aegypti*.

## Author Summary

Pyrethroids are one of the major classes of insecticides that is widely used to control mosquito vectors. The target site of pyrethroids is found in the voltage-sensitive Na^+^ channel (Vssc) consisting of about 2100 amino acid residues. In this study we generated several types of Vssc with a single or multiple mutations, expressed in *Xenopus* oocytes, and examined their electrophysiological properties using two-electrode voltage clamp method. We confirmed that *Aedes aegypti* Vssc harboring a triple mutations exhibited extremely high levels of resistance to pyrethroid insecticides. This Vssc type can be generated by a single crossing-over event of two resistant *Vssc* genes that are widely distributed in Southeast Asia, one of the greatest dengue endemic areas. Our results highlight the importance of intensive monitoring for the triple mutations in *Vssc* in the *A. aegypti* mosquito.

## Introduction

*Aedes aegypti* is the major mosquito vector of dengue fever (DF), yellow fever, and chikungunya fever. DF causes more illness and death in humans than any other arboviral disease [1]. Mosquito control is mainly achieved by pyrethroids because of the high and rapid activity of this class of insecticides in insects, and its low toxicity to mammals. However, intensive and frequent use of these chemicals has resulted in the development of resistance in *A. aegypti* [2], [3].

Pyrethroid insecticides target the voltage-sensitive Na^+^ channel (Vssc) [4]. Some amino acid substitutions alter the affinity of Vssc for pyrethroids and they confer resistance to this class of insecticide in insects [4]. Understanding how mutations alter interaction of pyrethroids with Vssc is important for improving our understanding of the pyrethroid mechanism of action and for constructing appropriate control strategies against pest insects. Previous studies have shown that additional amino acid substitutions concomitantly occurring in Vssc can further reduce an insect's susceptibility to pyrethroids. For instance, houseflies harboring Vssc with the L1014F substitution cause reduction in the affinity of Vssc for pyrethroids (so-called knockdown resistance or kdr), while the double mutation L1014F+M918T further reduces the susceptibility of the insects to pyrethroids (so-called super-kdr) [5].

In Southeast Asia, one of the largest DF endemic areas, two major *Vssc* haplotypes confer pyrethroid resistance to *A. aegypti*, i.e., S989P+V1016G and F1534C. *A. aegypti* with these two haplotypes are widely and sympatrically distributed in this area [6]–[8]. Neurophysiological studies have revealed that V1016G and F1534C single mutations each confer resistance to pyrethroids [9]. The F1534C mutation has also been confirmed from another dengue vector, *Aedes albopictus* [10]. S989P is usually accompanied by V1016G [11], but its contribution to pyrethroid resistance is unclear. Vssc harboring triple amino acid substitutions (i.e., S989P+V1016G+F1534C) has not been recorded from a field population of *A. aegypti* although heterozygous form of these two haplotypes were confirmed [7], [8]. Such a haplotype, however, can be generated by a single crossing-over event of the above two major resistance *Vssc* haplotypes. Moreover, the sensitivity of such a triple-mutated Vssc to pyrethroids has not yet been investigated.

In this study, we analyzed the pyrethroid sensitivity of Vssc harboring triple substitutions, using a two-electrode voltage-clamp method, and we discussed the effect thereof on the control of the dengue mosquito vector by pyrethroids. We also investigated the synergistic effect of S989P on the sensitivity of Vssc harboring the V1016G mutation to pyrethroid insecticides.

## Methods

### Ethics statement

This study complied with the guidelines for animal experiments performed at the National Institute of Infectious Diseases, Japan. The protocols for the use of mice and frogs were approved by the Animal Use Committee of the National Institute of Infectious Diseases (registration number: 211031) and Nippon-soda Co. Ltd. (registration number: 046), respectively. Animal experiments were carried out in adherence to the Guidelines for Proper Conduct of Animal Experiments established by Science Council of Japan in 2006 and the Animals (Scientific Procedures) Act 1986 by the Parliament of the United Kingdom. All surgery on *Xenopus* frogs was conducted under benzocaine anesthesia and all efforts were made to minimize suffering.

### Mosquito strains

Two strains of *A. aegypti* were used: SMK, a laboratory reference strain susceptible to pyrethroid insecticides, and SP, originally collected from Singapore in 2009, which has developed a high level of resistance to permethrin (1650-fold in adult females) after permethrin selection for 10 generations [6]. Mosquitoes were reared as described previously [12].

*In vitro* metabolism and synergistic studies revealed that both reduced permethrin susceptibility and enhanced metabolic enzyme activity, via cytochrome P450 monooxygenases, confer the high level of resistance in strain SP [6]. Our previous study confirmed that this strain possesses both S989P and V1016G mutations in *Vssc* homozygously [6]. The full length of *Vssc* cDNA in the SP strain, however, has not yet been sequenced.

### Chemicals

Permethrin (purity>99.1%) and deltamethrin (purity>99.0%) were purchased from LGC Standards (Teddington, UK) and Wako Pure Chemical Industries, Ltd. (Osaka, Japan), respectively. The stock solutions of insecticides (100 mM) were prepared in dimethyl sulfoxide (DMSO). Working solutions were prepared in standard oocyte saline (SOS; 100 mM NaCl, 2 mM KCl, 1.8 mM CaCl_2_, 1 mM MgCl_2_, 5 mM HEPES, pH 7.6) immediately prior to experiments. The DMSO concentration in the final solution was <0.1% and it did not affect the function of Na^+^ channels in the experiments (data not shown).

### Sequencing full-length *Vssc* cDNA from SMK and SP strains

Total RNA was extracted from 10 three-day-old females using ISOGEN (Nippon Gene Co., Ltd., Tokyo, Japan). Genomic DNA was removed by digesting the total RNA samples with DNase I using TURBO DNase (Life Technologies Co., Carlsbad, Ca, USA). cDNA was synthesized using RNA and QT' primer (5′- CCAGTGAGCAGAGTGACGAGGACTCGTGCTCAAGCT_15_-3′) and reverse transcriptase ReverTra Ace (Toyobo, Osaka, Japan) according to the manufacturer's instructions. The full-length cDNAs of *A. aegypti Vssc* were amplified by PCR using PrimeStar GXL DNA polymerase (Takara Bio Inc., Shiga, Japan) and the primers aegSCF1 (5′-ATGACCGAAGACTCCGATTCGA-3′) and aegSCR2 (5′-TCAGACATCCGCCGATCGT-3′). Cycling conditions for PCR were as follows: 95°C for 2 min, followed by 35 cycles of each consisting of 98°C for 10 s, 60°C for 15 s, and 68°C for 6 min. Nested PCR was performed under the same conditions, but using the primers aegSCF3 (5′-ATGACCGAAGACTCCGATTCGATAT-3′) and aegSCR4 (5′-TCAGACATCCGCCGATCGTGAA-3′). PCR products were purified by agarose gel electrophoresis and inserted into the pTA2 plasmid using the TArget Clone -Plus system (Toyobo, Osaka, Japan). The insert sequence was confirmed using the following primers: T7b (5′-CGTAATACGACTCACTATAGGGC-3′), aegSCF5 (5′-GTCGAATCTACCGAGGTGAT), aegSCF9 (5′-TGGTGTTACGATCAGCTGGA-3′), aegSCR8 (5′-CAGCCCTCTTGGAAGTAGTA-3′), AaSCF3 (5′-GTGGAACTTCACCGACTTCA-3′), aegSCF7 (5′-GAGGCGTTCAATCGGATATC-3′), AaSCR21 (5′-GCAATCTGGCTTGTTAACTTG-3′), aegSCF10 (5′-CGTTTCAGCTCATCGAGAAC-3′), AaSCR8 (5′-TAGCTTTCAGCGGCTTCTTC-3′), AlSCR8 (5′-AACAGCAGGATCATGCTCTG-3′), aegSCR6 (5′-TGCCTGAACTTCACCCAGTT-3′), and T3b (5′-CGCAATTAACCCTCACTAAAGGGA-3′). Eight and six clones of full-length *Vssc* cDNAs from the SMK and the SP strains, respectively, were sequenced. Nucleotide polymorphisms that were confirmed only in a single clone were considered to reflect polymerase errors.

### Site-directed mutagenesis and cloning of *tipE* cDNA

**Figure 1:**
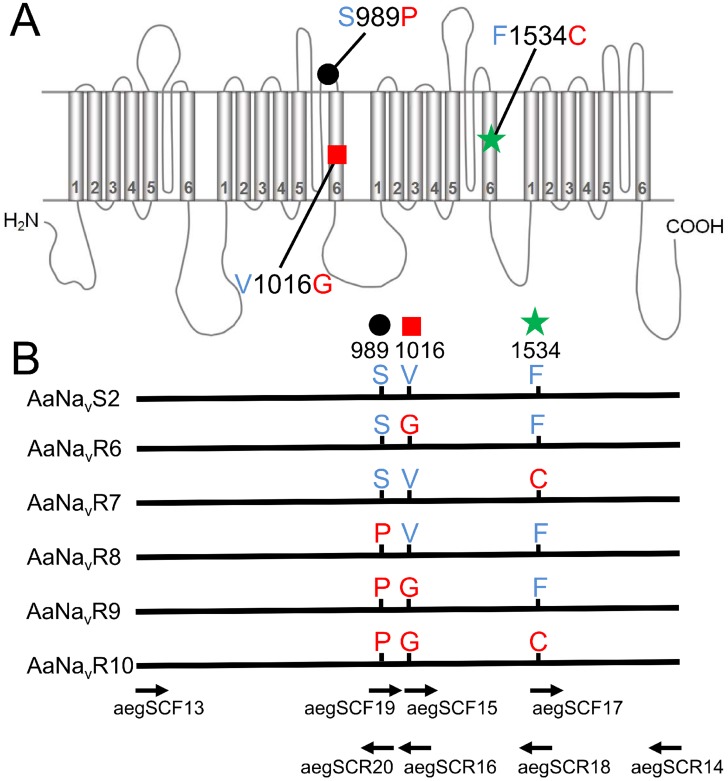
Mutations of *Aedes aegypti* Vssc investigated in this study. (A) Schematic illustration of the locations of the Vssc mutations assessed. Positions are numbered according to the amino acid sequence of the most abundant splice variant of housefly Vssc (GenBank accession nos. AAB47604 and AAB47605). (B) Six Vssc types investigated in this study and the positions of primers used for site-directed mutagenesis are shown. Primer sequences are listed in Methods. AaNa_v_S2 is the wild-type Vssc of which the cDNA was isolated from the insecticide-susceptible SMK strain.

Site-directed mutagenesis was performed by PCR using PrimeStar GXL DNA polymerase and primers that were designed based on the 5′ and 3′ end sequences of *A. aegypti Vssc* (*AaNa_v_S2*, DDBJ accession number: AB909019), which was isolated from insecticide susceptible SMK strain as described below. The forward primer aegSCF13 (5′-CGGAATTCATGACCGAAGACTCCGATTCGATAT-3′) and reverse primer aegSCR14 (5′-GAAGGCCTTCAGACATCCGCCGATCGTGAA-3′) included *Eco*RI and *Stu*I restriction sites (underlined), respectively, to facilitate cloning. Full-length cDNAs encoding the substitutions V1016G (*AaNa_v_R6*), F1534C (*AaNa_v_R7*), S989P (*AaNa_v_R8*), S989P+V1016G (*AaNa_v_R9*), and S989P+V1016G+F1534C (*AaNa_v_R10*) in *Vssc* were amplified, as shown in Figure 1. In this study we numbered the amino acid position according to the sequence of the most abundant splice variant of the house fly Vssc (GenBank accession nos. AAB47604 and AAB47605). Starting with *AaNa_v_S2* (wild-type), each fragment containing the smallest number of polymorphisms were subjected to oligonucleotide-mediated mutagenesis following a standard protocol [13], using the primers aegSCF15 (5′-GGAAATCTAGTAGGACTTAACCTTTTCTTAGC-3′), aegSCR16 (5′-GCTAAGAAAAGGTTAAGTCCTACTAGATTTCC-3′), aegSCF17 (5′-CTTCATCATCTGCGGGTCGTTCTT-3′), aegSCR18 (5′-AAGAACGACCCGCAGATGATGAAG-3′), aegSCF19 (5′-AGTGGATCGAACCCATGTGGGATT-3′), and aegSCR20 (5′-AATCCCACATGGGTTCGATCCACT-3′). Amplified PCR products were digested with restriction enzymes and cloned into the pTS1 vector (Nippon Gene Co., Ltd, Tokyo, Japan). Clones were fully sequenced and each amino acid substitution was confirmed. *Drosophila melanogaster* temperature-induced paralytic E (*tipE*) cDNA was also amplified and cloned into pcDNA3.1(+) (Invitrogen, Carlsbad, CA, USA) using published primer sequences [14]. The compatibility of *Drosophila* tipE with Vssc of other insects has been well documented [15]–[18].

### Preparation of cRNA

Templates for *in vitro* transcription were PCR-amplified from plasmids using KOD plus Neo (Toyobo) and the primers pTS1_cRNA_F (5′-GCGCATAATACGACTCACTATAGGGAG-3′) and pTS1_cRNA_R (5′-AGCTGTGCGCGCAAATTAACCCTC-3′). Capped RNA transcripts were synthesized using the mMessage mMachine T7 Ultra Kit (Life Technologies, Carlsbad, CA, USA). The quantity and quality of cRNAs were verified by agarose gel electrophoresis and absorption spectroscopy, and cRNAs were stored at −80°C until required for use.

### Expression of *A. aegypti* Vssc in *Xenopus* oocytes

Female *Xenopus laevis* were purchased from Hamamatsu Seibutsukyozai (Shizuoka, Japan) and maintained in dechlorinated water at 18°C. Oocytes were surgically obtained following anesthesia of a *Xenopus* frog using 0.03% benzocaine (Sigma-Aldrich, Tokyo, Japan), and enzymatically defolliculated by incubating them for 1–1.5 h in a Ca^2+^-free SOS containing 1 mg/ml collagenase (Wako Pure Chemical Industries, Osaka, Japan). *Vssc* cRNA and *tipE* cRNA (0.9 ng each) were co-injected into healthy stage V–VI oocytes, which were incubated at 18°C in SOS medium supplemented with 50 µg/ml gentamycin, 100 U/ml penicillin, 100 µg/ml streptomycin, 2.5 mM Na pyruvate, and 4% horse serum, for 2–4 days after injection. The medium was replaced daily and unhealthy oocytes were discarded. It is generally recognized that the knockdown resistance is a recessive trait [19]–[21]. Since we injected mRNA of a single Vssc haplotype into *Xenopus* oocytes, theoretically we reproduced the environment of the homozygous Vssc form in the cell.

### Electrophysiological recording environment

Na^+^ currents were recorded using a TEV-200A (Dagan Corporation, Minneapolis, MN, USA) amplifier and digitized at 40 kHz with PowerLab 8/30 (AD Instruments, Nagoya, Japan). Signals were filtered at 2 kHz using a Bessel four-pole filter (Dagan Corporation, Minneapolis, MN, USA). The P/N method was used to subtract capacitive transient currents [22]. Recording electrodes were prepared from borosilicate glass tubes (GC150TF-10; Warner Instruments, Hamden, CT, USA) using a P-1000 puller (Sutter Instruments, Novato, CA, USA). Electrodes were filled with 3 M KCl and had a resistance of 0.5–2.0 MΩ when measured in SOS. Oocytes were continuously perfused with SOS throughout the recording session, at a rate of 5 ml/min using a gravity-fed system. Experiments were performed at 18–19°C.

### Voltage protocols and data analysis

Methods for Na^+^-current recordings were performed using standard two-electrode voltage clamping, as described by Du et al. [17]. Na^+^ currents were elicited by a 20-ms test pulse from a holding potential of −120 mV to −20 mV. The peak current ensuing from 20-ms step depolarization, in response to a test potential (V) in the range of −80 mV to +40 mV, in 5-mV increments, from a holding potential, at 10-s intervals was measured. The voltage dependence of activation was then calculated by dividing by (V – V_rev_), where V_rev_ is the reversal potential as determined from the current-voltage curves. Peak conductance values were plotted against V and fitted with the Boltzmann equation to obtain the half-maximal activation voltage.

The voltage dependence of Vssc inactivation was determined using a 200-ms pre-pulse in the range of −100 mV to 0 mV, in 5 mV increments, followed by a 20-ms test pulse at −20 mV. Data were plotted as normalized current vs. inactivation pre-pulse voltage, and fitted with the Boltzmann equation to obtain the half-maximal inactivation voltage.

Application of permethrin and deltamethrin were conducted using the disposable perfusion system [23]. Pyrethroid-induced tail currents were recorded during a 100-pulse train of 5-ms depolarizations from a holding potential (−120 mV) to −20 mV at 10-ms intervals [17]. The amplitude of tail currents was used to establish the percentage of modified channels, as previously described [5], [16], [23], [24]. The decays of tail currents were fitted with single functions to determine the time constant (Tau values). The sensitivity of Vssc to each insecticide was defined as the concentration of insecticide at which 25% of channels were modified (EC_25_).

### Statistical analysis

The recorded data were analyzed using GraphPad Prism 5.04 software (GraphPad Software, Inc., La Jolla, CA, USA). Voltage dependences (both activation and inactivation of V_1/2_) were compared statistically between wild-type and mutant channels using one-way ANOVA followed by Dunnett's multiple comparison test. The decays of tail currents were fitted with single functions to determine time constants (Tau values). Statistical comparisons (one-way ANOVA followed by Dunnett's multiple comparison test) were made for time constants of the decays between the wild-type and the mutant channels at P<0.05. Tukey-Kramer HSD (Honestly significant difference) procedure was used to test for the differences of % modified channels among Vssc haplotypes after the treatment of insecticides.

## Results

### Cloning and sequencing the full-length *Vssc* cDNA from *A. aegypti*

Eight clones of full-length *Vssc* cDNA obtained from a pyrethroid-susceptible SMK strain were sequenced. The *Vssc* cDNA (six clones) of SP was also sequenced to determine if S989P and V1016G were the only substitutions present in Vssc in this strain. The SMK strain harbored two polymorphisms that both generated a substitution at amino acid 427 (R or K). K427 was not confirmed in the Vssc of strain SP. It is known that Vsscs have two mutually exclusive alternative exons (c and d) [25]; the expression of both exons was confirmed in both the SMK and SP strains. These two exons are 54 amino acids long and differ at two residues (V922+T952 and M922+I952 for exons c and d, respectively). In the SMK strain, the ratio of exon c and d were 0.54 and 0.46 (based on 13 clones), and we therefore concluded that these polymorphisms were not responsible for pyrethroid resistance. For electrophysiology studies, we used SMK-derived AaNa_v_S2 (DDBJ accession number: AB909019, consisted of 2109 amino acid residues), which contains R427 and exon c, as does the wild-type Vssc. We also confirmed that S989P and V1016G are the only amino acid substitutions present in the full-length *Vssc* cDNA in the SP strain.

### Functional analysis of AaNa_v_S2 in *Xenopus* oocytes

**Figure 2:**
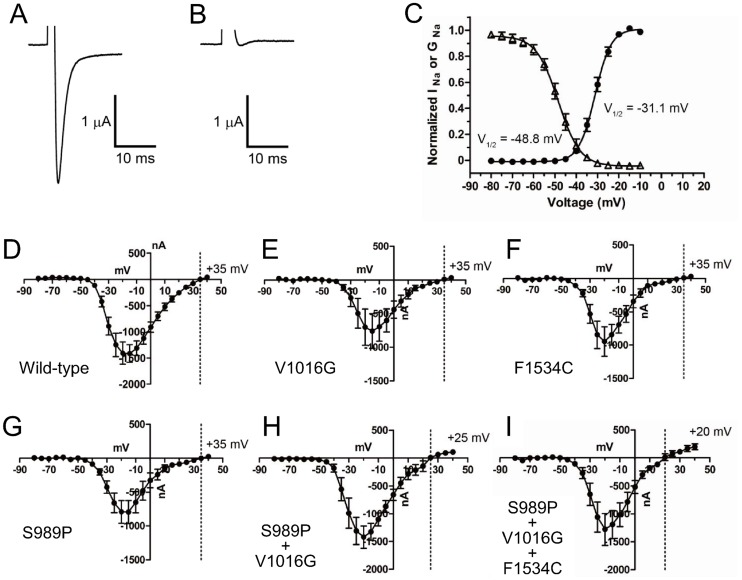
Structural and functional properties of *Aedes aegypti* Vsscs expressed in *Xenopus* oocytes. (A) The cloned AaNa_v_S2 (wild-type) channel was expressed in *Xenopus* oocytes and a Na^+^ current trace was recorded. (B) The Na^+^ current was blocked by the application of 10 nM tetrodotoxin, verifying that this was a Na^+^ current. (C) Normalized voltage-conductance and inactivation curves of AaNa_v_S2. The peak current was plotted against the depolarizing voltage (solid circles). The peak current amplitude was plotted as a function of the pre-pulse potential (triangles). Error bars indicate standard errors for 7–9 oocytes (D–I). Na^+^ current-voltage curves for 6 Vssc types. Error bars indicate standard errors for 7–9 oocytes. Reversal potentials were: (D) wild-type (+35 mV); (E) V1016G single mutation (+35 mV); (F) F1534C single mutation (+35 mV); (G) S989P single mutation (+35 mV); (H) S989P+V1016G double mutation (+25 mV); and (I) S989P+V1016G+F1534C triple mutation (+20 mV).

The wild-type *A. aegypti* Vssc (AaNa_v_S2) and *Drosophila* tipE, which is required for robust expression of insect Vssc, were co-expressed in *Xenopus* oocytes. No peak current was detected in oocytes that had not been injected with cRNA (data not shown). A peak current of approximately 0.5–3 µA was detected on days 2–4 in most oocytes that had been co-injected with wild-type *AaNa_v_S2* and *tipE* cRNA. A representative trace is shown in Figure 2A. Currents were completely inhibited by 10 nM tetrodotoxin (Figure 2B), confirming that they were Na^+^ currents.

Oocytes were clamped at a holding potential of −120 mV, then depolarized to test potentials from −80 mV to +40 mV in 5-mV increments (Figure 2C). Na^+^ currents through AaNa_v_S2 were detected at approximately −40 mV, and peaked at −15 mV (Figure 2C). The half-maximal activation voltage of wild-type AaNa_v_S2 was −31.1±0.4 mV and the slope factor was 3.9±0.3 mV (Table 1). To assess the voltage dependence of steady-state inactivation, oocytes were held at −120 mV and depolarized with a series of 200-ms inactivating pre-pulses, from −100 mV to 0 mV in 5-mV increments, followed by a 20-ms test pulse at −20 mV. The half-maximal inactivation voltage was −48.8±0.5 mV and the slope factor was 5.5±0.4 mV (Table 1, Figure 2C).

**Table 1:** Voltage-dependence of activation and inactivation of *Aedes aegypti* sodium channels and sensitivity of mutant channels to pyrethroids.

	Voltage dependence						Pyrethroid susceptibility					
Vssc type (mutations)	Activation			Inactivation			Permethrin			Deltamethrin		
	n^a^	V_1/2_ (mV)	*k* (mV)	n^a^	V_1/2_ (mV)	*k* (mV)	n^a^	EC_25_ (µM)	Ratio	n^a^	EC_25_ (µM)	Ratio
AaNa_v_S2 (Wild-type)	8	−31.1±0.4	3.9±0.3	7	−48.8±0.4	5.5±0.4	4	0.002	-	4	0.01	-
AaNa_v_R6 (V1016G)	7	−27.5±0.7*	4.2±0.5	8	−47.1±0.6	4.6±0.5	5	0.2	100	8	0.02	2
AaNa_v_R7 (F1534C)	7	−28.7±0.3*	4.0±0.2	7	−49.2±0.9	5.4±0.8	4	0.05	25	4	0.01	1
AaNa_v_R8 (S989P)	7	−28.4±0.4*	4.3±0.3	8	−48.5±0.4	3.9±0.4	4	0.002	1	4	0.01	1
AaNa_v_R9 (S989P+V1016G)	8	−32.0±0.4	3.5±0.4	7	−47.3±0.5	4.6±0.5	4	0.2	100	4	0.1	10
AaNa_v_R10 (S989P+V1016G+F1534C)	9	−29.4±0.8	5.0±0.7	7	−47.8±0.7	4.9±0.6	4	2.2	1100	4	0.9	90

Data were fitted with the Boltzmann equation to determine V_1/2_, the voltage of half-maximal conductance or inactivation, and *k*, the slope factor. Values represent the mean ± standard errors. Voltage dependences were compared statistically between wild-type and mutant channels using one-way ANOVA followed by Dunnett's multiple comparison test.

*P<0.05. Ratios were calculated by EC_25_ (mutant)/EC_25_ (wild-type).

a n, number of biological replications for each insecticide concentration tested.

### Effect of Vssc mutations on gating properties of channels

The expression of all five modified Vssc was confirmed in *Xenopus* oocytes. The effects of three substitutions (S989P, V1016G, and F1534C), alone or in combination, on the voltage dependence of activation, steady-state inactivation, and reversal potential were evaluated. Na^+^ currents recorded in oocytes expressing mutant channels exhibited voltage-dependent activation and inactivation, similar to those expressing the wild-type AaNa_v_S2 (Figures 2D–I, Table 1). The reversal potentials of AaNa_v_R9 (S989P+V1016G) and AaNa_v_R10 (S989P+V1016G+F1534C) mutants were slightly shifted, from +35 mV in the wild-type (Figure 2D) to +25 mV (Figure 2H) and +20 mV (Figure 2I) in the respective mutants. Three mutant channels, AaNa_v_R6 (V1016G), AaNa_v_R7 (F1534C), and AaNa_v_R8 (S989P) significantly (*P*<0.05 by one-way ANOVA followed by Dunnett's multiple comparison test) shifted the voltage-dependence of activation in the depolarizing direction (Table 1). None of the Vssc mutants changed the voltage dependence of inactivation significantly (Table 1).

### Effects of amino acid substitutions on the sensitivity of Vssc to permethrin

**Figure 3:**
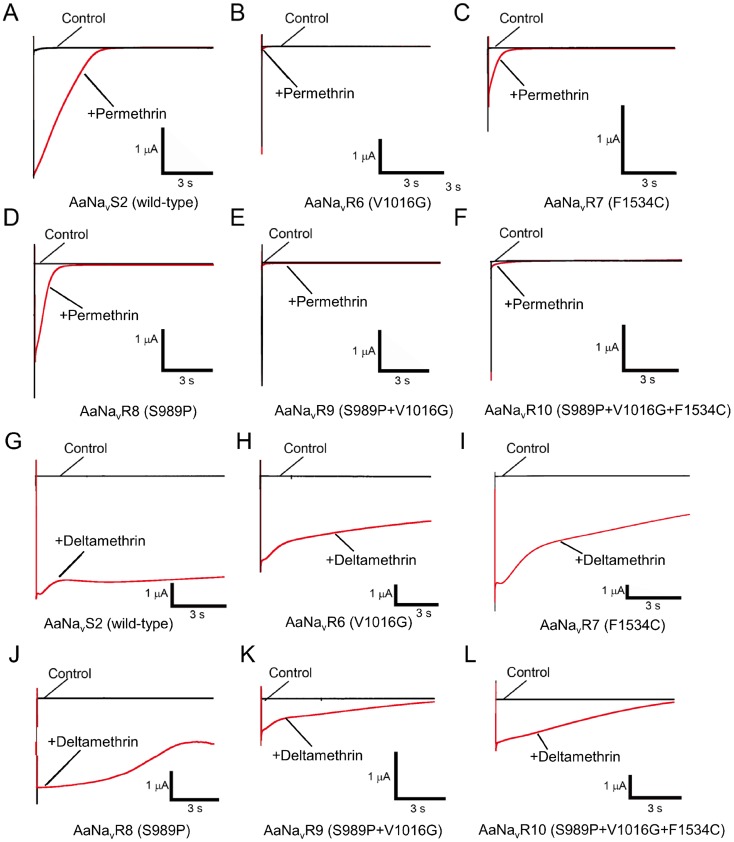
Pyrethroid-induced tail currents from oocytes injected with various types of Vssc. (A, G) AaNa_v_S2 (wild-type); (B, H) AaNa_v_R6 (V1016G); (C, I) AaNa_v_R7 (F1534C); (D, J), AaNa_v_R8 (S989P); (E, K), AaNa_v_R9 (S989P+V1016G); (F, L) AaNa_v_R10 (S989P+V1016G+F1534C) in the absence (control) or presence of 100 nM permethrin (A–F) or 100 nM deltamethrin (G–L).

Pyrethroid-induced tail currents were elicited with a train of 100 depolarizing pulses [5], [16], [23], [24]. Currents were recorded 10 min after application of permethrin (Figure 3). In the wild-type channel AaNa_v_S2, 100 nM permethrin induced large tail currents that recovered to baseline values within 3 s (Figure 3A). In all five mutant channels, the same concentration of permethrin induced only a small tail current that quickly recovered to baseline (Table 2, Figures 3B–F).

**Table 2:** Time constants of the decay (τ_decay_) of pyrethroid-induced tail currents.

Vssc type	Mutations	Permethrin	Deltamethrin
		τ_decay_ (s)	τ_decay_ (s)
AaNa_v_S2	Wild-type	2.0±0.2	9.7±1.5
AaNa_v_R6	V1016G	0.15±0.01*	3.6±1.1*
AaNa_v_R7	F1534C	0.33±0.10*	3.3±0.3*
AaNa_v_R8	S989P	0.60±0.08*	4.0±0.4*
AaNa_v_R9	S989P+V1016G	0.22±0.13*	3.8±2.0*
AaNa_v_R10	S989P+V1016G+F1534C	0.21±0.02*	N. D.

The decays of tail currents were fitted with single functions to determine time constants (Tau values). Values represent the mean ± standard errors. Statistical comparisons (one-way ANOVA followed by Dunnett's multiple comparison test) were made for time constants of the decays between the wild-type and the mutant channels at *P<0.05. N.D., not determined because the tail current tended to keep recovering to base line.

**Figure 4:**
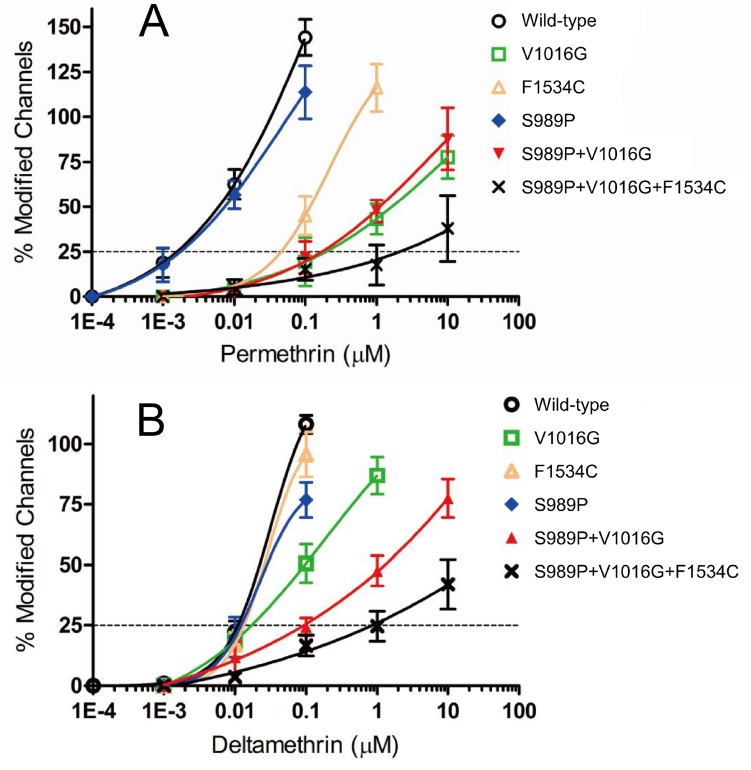
Sensitivity of *Aedes aegypti* Vsscs to permethrin and deltamethrin. The percentages of modified channels were plotted against different concentrations of permethrin (A) and deltamethrin (B), and fitted to a four-parameter logistic equation. Error bars indicate standard errors for 4–8 oocytes. The percentages of modified channels were significantly different between AaNa_v_R6 (V1016G) and AaNa_v_R9 (S989P+V1016G) at deltamethrin concentration of 1.0 µM (*P* = 0.0091 by Tukey-Kramer test) in Figure 4B.

The S989P mutation had no effect on permethrin sensitivity on its own (AaNa_v_R8), or in combination with V1016G (AaNa_v_R9) (Figure 4A). The V1016G and F1534C substitutions independently reduced sensitivity of Vssc to permethrin by 100- and 25-fold, respectively (Table 1, Figure 4A). The combination of three substitutions, viz., S989P+V1016G+F1534C (AaNa_v_R10), exhibited 1100-fold more resistance, which represents an 11-fold reduction (1100/100) and a 44-fold (1100/25) reduction in Vssc sensitivity to permethrin as compared to Vssc harboring S989P+V1016G or F1534C, respectively. This suggested that these substitutions acted synergistically (Table 1, Figure 4A).

### Effects of amino acid substitutions on the susceptibility of Vssc to deltamethrin

Deltamethrin-induced tail currents initially increased, and then gradually recovered in the wild-type channel (Figure 3G). However, the current in AaNa_v_R10 (S989P+V1016G+F1534C) had a lower amplitude and recovered to baseline more rapidly than did the wild-type (Figure 3L). As compared with wild-type Vssc, deltamethrin-induced tail currents of four mutant channels (AaNa_v_R6, -R7, -R8, and -R9) decayed very quickly (Table 2, Figures 3H–K). We could not estimate the actual time constant for AaNa_v_R10 (S989P+V1016G+F1534C) because the tail current tended to keep recovering to baseline. The deltamethrin-induced tail current of AaNa_v_R10, however, clearly decayed faster than that of the wild-type.

V1016G reduced the sensitivity of Vssc to deltamethrin by 2-fold, while F1534C alone exerted no effect on Vssc sensitivity (Table 1, Figure 4B). S989P alone (AaNa_v_R8) had no effect on deltamethrin sensitivity, but the combination of S989P and V1016G (AaNa_v_R9) reduced deltamethrin-sensitivity of Vssc by 10-fold (Table 1), which was a 5-fold reduction in deltamethrin-sensitivity over that induced by V1016G alone. The percentage of modified channels were significantly different between AaNa_v_R6 and AaNa_v_R9 at deltamethrin concentration of 1.0 µM (*P* = 0.0091 by Tukey-Kramer test). Furthermore, S989P+V1016G+F1534C (AaNa_v_R10) exhibited a 90-fold greater resistance than wild-type. This represents a 9-fold (90/10) and 90-fold (90/1) greater reduction in sensitivity than that induced by S989P+V1016G and F1534C, respectively, indicating a strong synergistic effect of the triple substitutions (Table 1). The percentage of modified channels were significantly different between AaNa_v_R9 and AaNavR10 at deltamethrin concentration of 10 µM (*P* = 0.034).

## Discussion

To date, the V1016G mutation (usually accompanied by S989P) of Vssc has been confirmed only in *A. aegypti* distributed in Southeast Asia, including Thailand [7], [11], Vietnam [8], Taiwan [26], Singapore [6], and the Philippines (Yukiko Higa, personal communications). F1534C mutation of Vssc has also been confirmed from all of above regions except for Taiwan [6]–[8]. These two haplotypes were confirmed as heterozygous form as well [7], [8]. A previous study revealed that Vssc harboring V1016G exhibited a greater reduction in permethrin susceptibility than did Vssc harboring F1534C, which was in agreement with our findings. Surprisingly, the combination of S989P, V1016G, and F1534C altered the sensitivity of the S989P+V1016G mutant to permethrin and deltamethrin by 11-fold (1100/100) and 9-fold (90/10), respectively. Theoretically, this haplotype can be generated as the result of a single crossing-over event involving two resistant haplotypes (i.e., S989P+V1016G and F1534C), which are widely and sympatrically distributed throughout Southeast Asia. Pyrethroid insecticides may lose their efficacy in the control of *A. aegypti* if such a new type of Vssc emerges in the field population of this mosquito species. Therefore, we strongly emphasize the importance of surveying of the triple mutations in *Vssc* in the field population of this key mosquito vector. Early detection of this unique haplotype may assist in vector control strategies by providing early warning of insecticide insensitivity.

The likelihood of occurrence of such a Vssc with triple mutations may depend on its fitness cost. In this study, we observed that the reversal potential of AaNa_v_R10 (S989P+V1016G+F1534C) shifted slightly, although there were no statistically significant differences in the voltage dependence of activation or in the voltage dependence of steady-state inactivation between AaNa_v_R10 and the wild-type AaNa_v_S2. In North and South America, where no V1016G mutation has been confirmed in the Vssc of *A. aegypti* to date, V1016I is widely distributed [27]–[29]. The double mutations V1016I+F1534C have already been detected in some of these regions [30] and this fact implies the possibility that S989P+V1016G+F1534C triple mutations can also emerge. According to a neurophysiological study, V1016I does not alter the susceptibility of Vssc to pyrethroid [9] while in another study quantitative trait loci (QTL) that affects permethrin resistance was mapped close to the *Vssc* gene with this mutation [31]. Anyway, the synergistic effect of V1016I on the pyrethroid-susceptibility of Vssc with F1534C is intriguing. In our study, we focused on characterization of triple mutations that can be generated by a single crossing-over event between two major resistant *Vssc* haplotypes, but the pyrethroid-susceptibility of Vssc harboring V1016G+F1534C or S989P+F1534C double mutations may also elucidate the synergistic effects of these mutations.

Our results exhibited that S989P has a synergistic effect on the sensitivity of V1016G-harboring Vssc to deltamethrin, but not to permethrin (Figure 4). This result contradicts with an earlier observation that S989P does not synergize with V1016G [9]. The discrepancy between these two reports may be due to the use of different evaluation methods. We measured the Na^+^ currents of each Vssc at five concentrations of deltamethrin, with at least 4 independent replications, and used EC_25_ values as a measure of sensitivity. The percentages of modified channels were significantly different between AaNa_v_R6 (V1016G) and AaNa_v_R9 (S989P+V1016G) at deltamethrin concentration of 1.0 µM (*P* = 0.0091 by Tukey-Kramer test; Figure 4B). In contrast, previous studies used the ratios of modified channels at only one concentration of the chemical; it is possible that this may have led to an underestimation of the effect of S989P. Our findings highlight the need for genotyping S989P mutation as well as V1016G and F1534C for better understanding the pyrethroid susceptibility of the field population of *A. aegypti* collected especially in Southeast Asia.

## References

[1] Gubler DJ (1998) Dengue and dengue hemorrhagic fever. Clin Microbiol Rev 11: 480–496. 9665979 10.1128/cmr.11.3.480 PMC88892

[2] Hemingway J, Hawfes NJ, McCarroll L, Ranson H (2004) The molecular basis of insecticide resistance in mosquitoes. Insect Biochem Mol Biol 34: 653–665. 15242706 10.1016/j.ibmb.2004.03.018

[3] Vontas J, Kioulos E, Pavlidi N, Morou E, della Torre A, et al (2012) Insecticide resistance in the major dengue vectors *Aedes albopictus* and *Aedes aegypti* . Pestic Biochem Physiol 104: 126–131.

[4] Davies TGE, Field LM, Usherwood PNR, Williamson MS (2007) DDT, pyrethrins, pyrethroids and insect sodium channels. IUBMB Life 59: 151–162. 17487686 10.1080/15216540701352042

[5] Vais H, Williamson MS, Goodson SJ, Devonshire AL, Warmke JW, et al (2000) Activation of *Drosophila* sodium channels promotes modification by deltamethrin. Reductions in affinity caused by knock-down resistance mutations. J Gen Physiol 115: 305–318. 10694259 10.1085/jgp.115.3.305 PMC2217214

[6] Kasai S, Komagata O, Itokawa K, Shono T, Ng LC, et al (2014) Mechanisms of pyrethroid resistance in the dengue mosquito vector, *Aedes aegypti*: target site insensitivity, penetration, and metabolism. PLoS Negl Trop Dis 8: e2948. 24945250 10.1371/journal.pntd.0002948 PMC4063723

[7] Stenhouse SA, Plernsub S, Yanola J, Lumjuan N, Dantrakool A, et al (2013) Detection of the V1016G mutation in the voltage-gated sodium channel gene of *Aedes aegypti* (Diptera: Culicidae) by allele-specific PCR assay, and its distribution and effect on deltamethrin resistance in Thailand. Parasit Vectors 6: 253. 24059267 10.1186/1756-3305-6-253 PMC3765916

[8] Kawada H, Higa Y, Komagata O, Kasai S, Tomita T, et al (2009) Widespread distribution of a newly found point mutation in voltage-gated sodium channel in pyrethroid-resistant *Aedes aegypti* populations in Vietnam. PLoS Negl Trop Dis 3: e527. 19806205 10.1371/journal.pntd.0000527 PMC2754656

[9] Du Y, Nomura Y, Satar G, Hu Z, Nauen R, et al (2013) Molecular evidence for dual pyrethroid-receptor sites on a mosquito sodium channel. PNAS 110: 11785–11790. 23821746 10.1073/pnas.1305118110 PMC3718148

[10] Kasai S, Ng LC, Lam-Phua SG, Tang CS, Itokawa K, et al (2011) First detection of a putative knockdown resistance gene in major mosquito vector, *Aedes albopictus* . Jpn J Infect Dis 64: 217–221. 21617306

[11] Srisawat R, Komalamisra N, Eshita Y, Zheng M, Ono K, et al (2010) Point mutations in domain II of the voltage-gated sodium channel gene in deltamethrin-resistant *Aedes aegypti* (Diptera:Culicidae). Appl Entomol Zool 45: 275–282.

[12] Kasai S, Weerasinghe IS, Shono T (1998) P450 monooxygenases are an important mechanism of permethrin resistance in *Culex quinquefasciatus* Say larvae. Arch Insect Biochem Physiol 37: 47–56.

[13] Ho SN, Hunt HD, Horton RM, Pullen JK, Pease LR (1989) Site-directed mutagenesis by overlap extension using the polymerase chain reaction. Gene 77: 51–59. 2744487 10.1016/0378-1119(89)90358-2

[14] Feng G, Deak P, Chopra M, Hall LM (1995) Cloning and functional analysis of tipE, a novel membrane protein that enhances drosophila *para* sodium channel function. Cell 82: 1001–1011. 7553842 10.1016/0092-8674(95)90279-1

[15] Hu Z, Du Y, Nomura Y, Dong K (2011) A sodium channel mutation identified in *Aedes aegypti* selectively reduces cockroach sodium channel sensitivity to type I, but not type II pyrethroids. Insect Biochem Mol Biol 41: 9–13. 20869441 10.1016/j.ibmb.2010.09.005 PMC3022105

[16] Burton MJ, Mellor IR, Duce IR, Davies TGE, Field LM, et al (2011) Differential resistance of insect sodium channels with *kdr* mutations to deltamethrin, permethrin and DDT. Insect Biochem Mol Biol 41: 723–732. 21640822 10.1016/j.ibmb.2011.05.004

[17] Du Y, Nomura Y, Luo N, Liu Z, Lee JE, et al (2009) Molecular determinants on the insect sodium channel for the specific action of type II pyrethroid insecticides. Toxicol Appl Pharmacol 234: 266–272. 19022275 10.1016/j.taap.2008.10.006 PMC3052259

[18] Tan J, Liu Z, Wang R, Huang ZY, Chen AC, et al (2004) Identification of amino acid residues in the insect sodium channel critical for pyrethroid binding. Mol Pharmacol 67: 513–522. 15525757 10.1124/mol.104.006205

[19] Shono T (1985) Pyrethroid resistance: importance of the *kdr*-type mechanism. J Pestic Sci 10: 141–146.

[20] Huang J, Kristensen M, Qiao CL, Jespersen JB (2004) Frequency of *kdr* gene in house fly field populations: correlation of pyrethroid resistance and *kdr* frequency. J Econ Entomol 97: 1036–1041. 15279288 10.1093/jee/97.3.1036

[21] Gao JR, Yoon KS, Lee SH, Takano-Lee M, Edman JD, et al (2003) Increased frequency of the T929I and L932F mutations associated with knockdown resistance in permethrin-resistant populations of the human head louse, *Pediculus capitis*, from California, Florida, and Texas. Pestic Biochem Physiol 77: 115–124.

[22] Bezanilla F, Armstrong CM (1977) Inactivation of the sodium channel. I. Sodium current experiments. J Gen Physiol 70: 549–566. 591911 10.1085/jgp.70.5.549 PMC2228478

[23] Tatebayashi H, Narahashi T (1994) Differential mechanism of action of the pyrethroid tetramethrin on tetrodotoxin-sensitive and tetrodotoxin-resistant sodium channels. J Pharmacol Exp Ther 270: 595–603. 8071852

[24] Tan J, Liu Z, Tsai TD, Valles SM, Goldin AL, et al (2002) Novel sodium channel gene mutations in *Blattella germanica* reduce the sensitivity of expressed channels to deltamethrin. Insect Biochem Mol Biol 32: 445–454. 11886779 10.1016/s0965-1748(01)00122-9 PMC3057061

[25] Thackeray JR, Ganetzky B (1995) Conserved alternative splicing patterns and splicing signals in the Drosophila sodium channel gene *para* . Genetics 141: 203–214. 8536968 10.1093/genetics/141.1.203 PMC1206718

[26] Lin YH, Tsen WL, Tien NY, Luo YP (2013) Biochemical and molecular analyses to determine pyrethroid resistance in *Aedes aegypti* . Pestic Biochem Physiol 107: 266–276.

[27] Harris AF, Rajatileka S, Ranson H (2010) Pyrethroid resistance in *Aedes aegypti* from Grand Cayman. Am J Trop Med Hyg 83: 277–284. 20682868 10.4269/ajtmh.2010.09-0623 PMC2911171

[28] Aponte HA, Penilla RP, Dzul-Manzanilla F, Che-Mendoza A, López AD, et al (2013) The pyrethroid resistance status and mechanisms in *Aedes aegypti* from the Guerrero state, Mexico. Pestic Biochem Physiol 107: 226–234.

[29] Marcombe S, Mathieu RB, Pocquet N, Riaz MA, Poupardin R, et al (2012) Insecticide resistance in the dengue vector Aedes aegypti from Martinique: distribution, mechanisms and relations with environmental factors. PLoS One 7: e30989. 22363529 10.1371/journal.pone.0030989 PMC3283601

[30] Linss JGB, Brito LP, Garcia GA, Araki AS, Bruno RV, et al (2014) Distribution and dissemination of the Val1016Ile and Phe1534Cys *Kdr* mutations in *Aedes aegypti* Brazilian natural populations. Parasit Vectors 7: 25. 24428880 10.1186/1756-3305-7-25 PMC3912884

[31] Saavedra-Rodriguez K, Strode C, Suarez AF, Salas IF, Ranson H, et al (2008) Quantitative trait loci mapping of genome regions controlling permethrin resistance in the mosquito *Aedes aegypti* . Genetics 180: 1137–1152. 18723882 10.1534/genetics.108.087924 PMC2567363

